# Immediate and Intermediate-Term Outcomes of Infants With Transposition of Great Arteries Who Underwent Balloon Atrial Septostomy in Sudan

**DOI:** 10.5334/gh.1387

**Published:** 2025-01-20

**Authors:** Sulafa K. M. Ali, Amna Elsheikh, Mohammed Abdulrahman Alhassan

**Affiliations:** 1 Sudan Heart Center, University of Khartoum, Sudan; 2 Al Buluk Hospital, Omdurman, Sudan; 3 Prince Sattam Bin Abdulaziz University, Al Kharj, Saudi Arabia

**Keywords:** Transposition of great arteries, balloon septostomy, outcomes, Sudan

## Abstract

Transposition of great arteries (TGA) is a critical congenital heart disease leading to a fatal outcome if timely management is not provided. Management in low-income countries is challenging.

A retrospective analysis was carried out at Sudan Heart Center for infants with TGA who underwent balloon atrial septostomy (BAS) from January 2010 to December 2020. Immediate clinical- and procedure-related outcomes were evaluated. Intermediate-term outcomes were studied using follow-up hospital records as well as direct telephone calls.

The study included 75 infants (70% males) with a median age at presentation of 25 and at the time of BAS of 28 days. Pre-BAS median oxygen saturation was 48 (Interquartile Range (IQR) 40–60%). BAS was performed under fluoroscopy and echocardiography guidance with immediate success achieved in 98% of patients. Post-BAS oxygen saturation increased to 87 (IQR 85–90%) with a median improvement of 40% (p = 0.048), which was more significant in those less than 2 weeks of age. Minor complications occurred in 14 patients, and two patients (2.6%) died. Surgery (atrial in 44% and arterial switch operations in 41%) was performed in 39 patients (52%) at a mean age of three months with perioperative mortality of 30%. Infants who underwent surgery had a significantly higher survival rate (69%) compared to those who did not (5.6%) (p < 0.001).

Patients with TGA present at a late age with good immediate outcomes of BAS. Access to surgery is limited with a high surgical mortality. Those who survived surgery had good intermediate-term outcomes while most unoperated patients died.

## Introduction

Congenital heart disease (CHD) is one of the most common causes of infant mortality all over the world, with an incidence of at least 1:100 liveborn neonates; out of these, about 20% are considered critical CHD with a fatal outcome in early infancy if not timely treated (1). It is estimated that the number of children born with CHD in sub-Saharan Africa is nearly threefold compared with that reported in the United States of America; over 90% of these children do not have access to cardiac care (2). Transposition of the great arteries (TGA) represents about 6% of all CHD with an incidence of 1:3,500–5,000 live births (3). It typically presents in the first few days of life with severe cyanosis; to survive, patients need to have a shunt to facilitate blood mixing between systemic and pulmonary parallel circulations. If there is no/small shunt, patients require urgent palliation with prostaglandin infusion, creation of an atrial septal defect by balloon atrial septostomy (BAS), and, ultimately, surgical correction. In developing countries, TGA management poses a great challenge as there is a delay in diagnosis and referral to cardiac centers. Prostaglandin is often not available, and there is deficiency in intensive care units where these babies need to be critically managed. Surgery is mostly not available in such settings; therefore, BAS is considered the only life-saving procedure. However, BAS needs an experienced operator and assisting team as well as special cardiac catheterization equipment. The first report of BAS in Africa was in 1971 by South African cardiologists (4). A report in 1990 from Abidjan, where BAS was performed in 11 patients, is probably one of the earliest reports of BAS in low-income African countries. In this report, the procedure was successful in 8/11 patients, with two mortalities (5). In Sudan, the situation for TGA management is comparable to low-income countries where PGE and surgery are not routinely available; however, interventional catheterization has been steadily growing since 2004 (6,7). This paper reports the immediate- and intermediate-term outcome of patients with TGA who underwent BAS in Sudan.

## Patients and Methods

This study is a retrospective review of all patients who underwent BAS at the Sudan Heart Center for TGA from January 2010 to December 2020. The center is one of three pediatric cardiac referral centers in Sudan, receiving annually an average of 800 patients referred from all over the country. There is one interventional pediatric cardiologist (the first author) and three noninvasive cardiologists with an average cardiac catheterization load of 200 cases/year. At the time of the study, there was no dedicated congenital heart surgeon. There are three congenital heart surgeons in the other two centers performing open-heart surgery for infants weighing more than five kilograms; however, patients with TGA and other complex CHD cannot be routinely operated on in Sudan and are sent abroad for surgery.

The data was collected from medical records and included demographic, clinical, and procedural information as well as outpatient follow-up. Patients who did not show for follow-up were contacted by telephone calls using a data sheet that includes surgical interventions, mortality, and functional class.

## BAS Procedure

BAS was performed under fluoroscopy and echocardiography guidance with sedation. The femoral vein was used in 95% and the umbilical vein in 5% of patients. The vein is punctured using a 2.5 cm long, 21-gauge puncture needle. Over a 0.021 inch wire, a 5 or 7 French sheath (according to the balloon catheter used) is inserted in the vein. The balloon catheter is advanced to the right atrium, then through the fossa ovalis to the left atrium under echocardiography guidance. The balloon is inflated with diluted contrast and abruptly pulled through the fossa ovalis to the right atrium, retracted from the inferior vena cava, and then deflated. The atrial communication is evaluated by echocardiography aiming to see a flap that indicates an adequate atrial septal defect. Until 2015,the balloons used were Miller-Edwards ^R^, and Z Balloon ^R^ has been used afterwards.

## Ethical Considerations

All the parents were counseled about the features of TGA and the BAS procedure, and informed consent was obtained for the BAS procedure. The protocol of the study was approved by the Sudan Medical Specialization Board Ethics Committee. Approval was obtained from the Sudan Heart Center administration. Consent was taken verbally from guardians of patients who were contacted by telephone.

## Statistical Methods

Data were analyzed using STATA 18 software (StataCorp. 2021. *Stata Statistical Software: Release 17*. College Station, TX: StataCorp LLC). Descriptive statistics for continuous variables were reported as medians and interquartile ranges (IQR) due to their non-normal distribution and as means and standard deviations (SD) where appropriate. Categorical variables were summarized using frequencies and percentages. The Mann–Whitney U test was employed to compare continuous variables between groups, while Fisher’s exact test and the Chi-squared test were used to assess associations between categorical variables. Kaplan-Meier survival analysis was performed to evaluate the survival distribution, and the log-rank test was used to compare survival curves between those who did and those who did not undergo surgery. A p-value of less than 0.05 was considered statistically significant.

## Results

**Figure 1:**
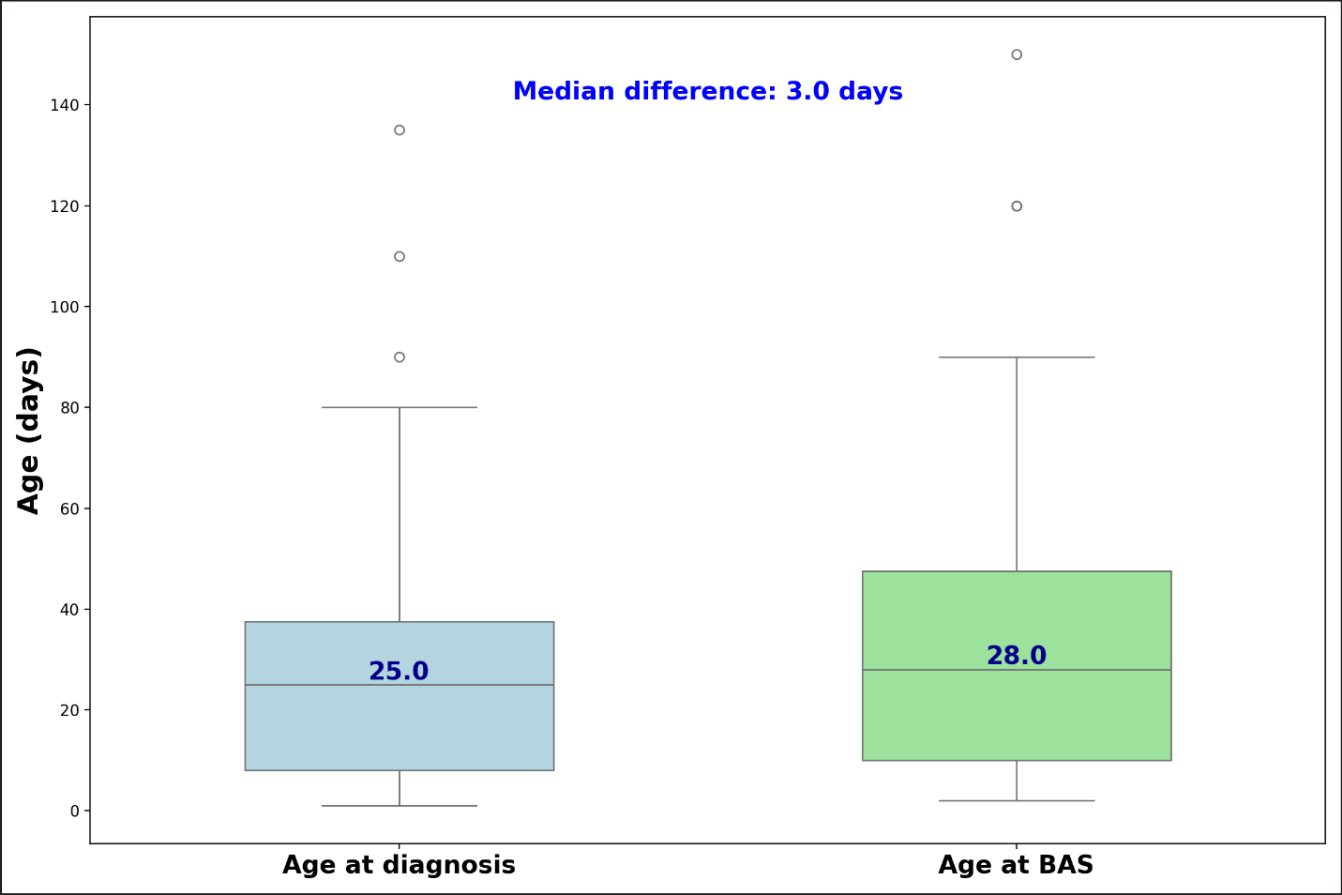
Timing of diagnosis and BAS in infants with TGA.

The study included 75 infants (70% males) with TGA who underwent BAS in Sudan. Clinical and procedural details are shown in Table 1. The median age at presentation was 25 (8–40) days, and at the time of BAS, it was 28 (10–50) days (Figure 1). The mean weight at the time of BAS was 3.2 +/– 0.5 kg. Pre-BAS median oxygen saturation was 48 (IQR 40–60)%. Associated defects included patent foramen ovale (66%), patent ductus arteriosus (15%), small atrial septal defect (12%), and small ventricular septal defect (10%).

**Table 1:** Demographic and clinical characteristics in relation to Survival at the last follow up.


CHARACTER	OUTCOME			P-VALUE

	ALIVE; 29 (39%)	DIED; 46 (61%)	TOTAL; 75 (100%)	

Age at diagnosis in days; median (Inter quaternary range IQR)	20 (6–30)	25 (10–40)	25 (8–40)	0.346

Age at the time of BAS in days (IQR)	26 (9–44)	30 (17–50)	28 (10–50)	0.424

Age at the time of corrective surgery in days; median (IQR)	90 (60–150)	150 (90–240)	90 (60–180)	0.057*

Weight at the time of BAS in kg; mean (SD)	3.3 (0.6)	3.1 (0.4)	3.2 (0.5)	0.075

Pre-BAS Oxygen Saturation (%); median (IQR)	40 (40–50)	50 (40–60)	48 (40–60)	0.049*

Post-BAS Oxygen Saturation (%); median (IQR)	87 (85–90)	87 (85–90)	87 (85–90)	0.326

Oxygen saturation (%) improvement after BAS; median (IQR)	45 (34–50)	35 (30–45)	40 (30–45)	0.048*

Corrective surgery done				<0.001*

No	2 (6%)	34 (94%)	36 (48%)	

Yes	27 (69%)	12 (31%)	39 (52%)	

Type of corrective surgery				

Arterial switch	13/39 (33 %)	3/39 (8%)	16/39 (41%) Primary arterial switch: 8 Staged arterial switch: 8	

Atrial switch	15/39 (38%)	2/39 (5%)	17/39 (44%)	

Unknown	0 (0%)	6/39 (15%)	6/39 (15%)	

Duration between BAS and corrective surgery in days; median (IQR)	60 (37–108)	107 (86–210)	67 (45–124)	0.012*


*Statistically significant.

## BAS Outcomes

**Figure 2:**
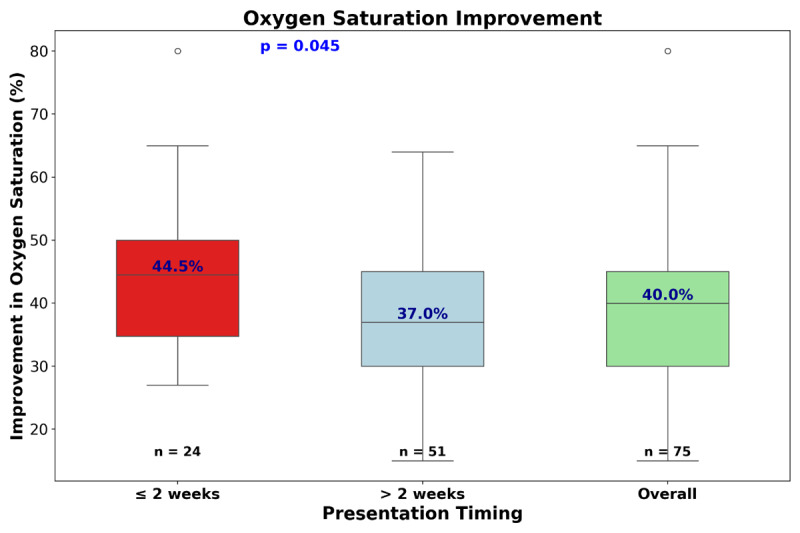
**Difference between pre and post BAS oxygen saturation (%)**: red: early presenters, blue: late presenters, green: all patients.

Immediate success was achieved in 74 cases (99%). Repeated BAS was successfully done in one more patient. Oxygen saturation improved significantly to 87 (IQR 85–90), compared with the pre-BAS level (p = 0.048); the median increment was 40 (IQR 30–45). There is a statistically significant difference in O2 saturation improvement post-BAS between early (≤ 2 weeks) and late (>2 weeks) presenters (p = 0.05) as shown in Figure 2. Minor complications during or immediately after BAS occurred in 14 patients (20%) and included transient arrhythmias(n = 8), bleeding (n = 5), and thrombosis (n = 1). Two patients died (2.6%); one went into cardiac arrest and was successfully resuscitated before BAS, then developed a second cardiac arrest and succumbed after the BAS. The second went into cardiac arrest immediately following BAS and could not be revived. After successful BAS, patients were advised to seek surgical correction, and they were followed up in the outpatient clinic.

## Surgery for TGA

**Figure 3:**
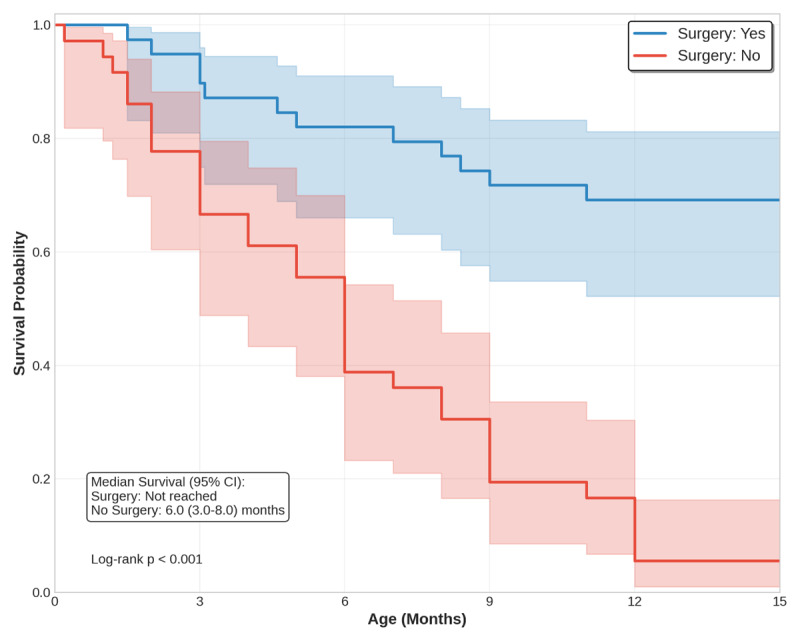
**Kaplan-Meier survival curves for infants with TGA**, comparing those who underwent corrective surgery (blue) and those who did not (orange). The survival probability was significantly higher in the surgery group (p < 0.001). Median survival is not reached for those who underwent surgery, as majority of infants in this group survived for the entire study duration.

Corrective surgery was performed in 39 patients (52%). Infants who underwent corrective surgery had a significantly higher survival rate (69%) compared to those who did not (5.6%) (p < 0.001) (Figure 3). The median duration between BAS and surgery was shorter for survivors than non-survivors (p = 0.012), as shown in Table 1. The type of surgery was atrial switch in 44% and arterial switch operation in 41%. The cause of delay in surgical correction was unavailability in Sudan in 95% of patients. Overall operative mortality was 30%. Thirty patients (70%) were operated on in country I, with a survival rate of 75%, while four were operated on in country E with a survival rate of 24%, and 2 patients were operated on in Sudan by a visiting team, and both survived. The three remaining patients were operated on in three other countries.

## Intermediate-term Outcomes

Follow-up data was available for all 75 patients. The mean duration of follow-up was 30 (12–150) months. Most patients who survived operations (97%) were asymptomatic. There were two patients who survived without surgery; both were eight years old with severe cyanosis and functional limitation.

## Discussion

This study described the immediate- and intermediate-term outcomes of a cohort of patients with TGA and documented a good immediate outcome of BAS despite limitations of management of this critical CHD in a low-income country. The median age at the time of BAS of 28 days is extremely late when compared with a study from the Netherlands where the median age of patients was 1 day, and most patients were diagnosed by fetal echocardiography. This variation in the age of diagnosis highlights the substantial lack of resources in low-income countries (8).

Many of our patients had small shunts that potentially improve mixing; these patients could probably be considered natural survivors, strikingly up to 150 days of age. We may speculate that there is a significant number of infants who died before reaching our center, as we encountered many patients with unstable hemodynamic status in this cohort. The decision to perform BAS in such patients is challenging, as mortality could be high during the procedure, as noted in our patients. This pattern of late presentation is attributed to many factors common to low-income countries, including late diagnosis due to deficient antenatal echocardiography services together with limited neonatal screening programs. Moreover, referral of suspected CHD is delayed due to limited pediatric cardiology services in remote areas, coupled with a shortage of neonatal and intensive care units and unavailability of prostaglandin. Pediatric cardiology services are often costly and not supported by medical insurance, leading to delays in seeking medical attention. Such limitations had also been reported, but to a lesser degree, in middle-income countries (9,10).

There are few reports about outcomes of TGA in African countries. One study from Nigeria in 2017 included 51 patients with TGA, with about 60% having intact ventricular septum; however, there were no reported cases where BAS was performed. Only 10% of patients underwent surgery, all of which were outside Nigeria, with only one survivor. Although this is far less than the intervention rate in our patients, we believe that this is the usual pattern for many low-income countries in Africa (11).

Despite late presentation and poor hemodynamic status, the immediate outcomes of BAS were good with a significant increase in oxygen saturation comparable with the recent study from The Netherlands (8). The improvement was more significant in early presenters stressing the importance of early diagnosis and management of patients with TGA. This finding could also be related to technical difficulties of creating adequate atrial communication in older infants.

Only over half of patients managed to have surgical repair, primarily because surgery is not available in Sudan, leading to expensive travel abroad. Surgical outcomes were remarkable for an operative mortality of 30%, which indicates limited surgical skills and perioperative facilities in the countries where surgery was performed. Poor outcomes could also be related to the old age at surgery, which adversely affected both the type of operation as well as the outcome in this cohort. There is a huge discrepancy between the surgical outcomes of our patients and those of the United States of America, where the reported operative mortality is 3% and the 25-year survival is over 96% (12). Our settings are comparable with many developing countries where surgery for CHD, particularly for TGA, is not available, leading to a huge burden of infant mortality. While the infant mortality from CHD has globally dropped by 34%, most mortalities were noted to occur in low- and middle-income countries (13). A study from 26 centers in developing countries (excluding Africa) that examined the postoperative outcomes of 480 patients with TGA and intact ventricular septum revealed a perioperative mortality of 15% despite that 50% of patients presented after the age of one month. This mortality rate is 50% less when compared with our patients, indicating that the situation in Africa is worse than in non-African developing countries. In the later study, the mortality was significantly related to small body weight and low institutional volume. This fact indicates the need to have regional referral pediatric cardiac centers with a large volume of patients that are accessible and affordable for less developed countries (14). Surgery for TGA is particularly challenging as it requires advanced technical skills as well as intensive care facilities, mostly not available in low-income countries. Few African centers started collaboration with developed countries aiming to improve the capacity building of African surgeons (15).

Correction at a later age significantly increased operative mortality in our patients and led to performance of more atrial than arterial switch operations. This is due to the fact that in infants older than 2–4 weeks, the pulmonary vascular resistance drops gradually, leading to a decrease in the left ventricle volume and pressure. This results in involution of the left ventricle and an inability to tolerate the systemic pressure when an arterial switch operation is performed. In this situation, either training of the left ventricle by a pulmonary artery band or doing an atrial switch operation are the only available surgical options. Although our patients who had atrial switch were doing well at intermediate-term followup, this operation is expected to have many complications in adulthood due to right ventricle failure; obviously, atrial switch is currently rarely performed in the era of arterial switch operation (16). Although the intermediate-term survival for those who had successful surgery was excellent, their neurological and developmental outcomes still need to be evaluated since they were exposed to prolonged hypoxia. Many patients who underwent surgery for TGA were found to have cognitive and psychomotor difficulties even when timely managed in centers of excellence (17).

Most of those who did not have surgery died by one year of age, emphasizing the poor outcome of unoperated children with TGA. This finding stands out as a sign of inequity in CHD management in developing countries and stresses the urgent need for action to improve this situation. Establishing regional cardiac centers of excellence coupled with early diagnosis and management through training of physicians and availability of antenatal and neonatal screening programs are all needed to improve the outcomes of children with CHD. These global gaps in CHD management were addressed at the 8^th^ World Congress of Pediatric Cardiology and Cardiac Surgery, where an advocacy statement was issued aligned with the 2030 Global Agenda for Sustainable Development. The main themes of this statement were improving capacity building, data availability, and financing (18).

In conclusion, we described the immediate- and intermediate-term outcomes of patients with TGA who underwent BAS at the Sudan Heart Center and documented a late age of presentation and favorable immediate outcomes. There is poor access to surgery and high operative mortality. Those who survived operations have good intermediate-term outcomes. Our data emphasize the need for improving early diagnosis of critical CHD and establishing regional centers of excellence in order to overcome inequities in CHD management.
